# Preparation and Characterization of Novel Mixed Periodic Mesoporous Organosilica Nanoparticles

**DOI:** 10.3390/ma13071569

**Published:** 2020-03-28

**Authors:** Hao Li, Laurence Raehm, Clarence Charnay, Jean-Olivier Durand, Roser Pleixats

**Affiliations:** 1 Department of Chemistry and Centro de Innovación en Química Avanzada (ORFEO-CINQA), Faculty of Sciences, Universitat Autònoma de Barcelona. UAB Campus, C/ dels Til.lers, 08193 Cerdanyola del Vallès, Spain; Hao.Li@uab.cat; 2 ICGM, CNRS, ENSCM, University of Montpellier, Case 1701, Place Eugène Bataillon, CEDEX 05, 34095 Montpellier, France; clarence.charnay@umontpellier.fr (C.C.); laurence.raehm@umontpellier.fr (L.R.)

**Keywords:** periodic mesoporous organosilica nanoparticles, sol-gel process, disilylated *tert*-butyl 3,5-dialkoxybenzoates

## Abstract

We report herein the preparation of mixed periodic mesoporous organosilica nanoparticles (**E-Pn 75/25** and **90/10 PMO NPs**) by sol-gel co-condensation of *E*-1,2-bis(triethoxysilyl)ethylene ((*E*)-BTSE or **E**) with previously synthesized disilylated *tert*-butyl 3,5-dialkoxybenzoates bearing either sulfide (precursor **P1**) or carbamate (precursor **P2**) functionalities in the linker. The syntheses were performed with cetyltrimethylammonium bromide (CTAB) as template in the presence of sodium hydroxide in water at 80 °C. The nanomaterials have been characterized by Transmission Electron Microscopy (TEM), nitrogen-sorption measurements (BET), Dynamic Light Scattering (DLS), zeta-potential, Thermogravimetric Analysis (TGA), FTIR, ^13^C CP MAS NMR and small angle X-ray diffraction (p-XRD). All the nanomaterials were obtained as mesoporous rodlike-shape nanoparticles. Remarkably, **E-Pn 90/10 PMO NPs** presented high specific surface areas ranging from 700 to 970 m^2^g^−1^, comparable or even higher than pure **E PMO** nanorods. Moreover, XRD analyses showed an organized porosity for **E-P1 90/10 PMO NPs** typical for a hexagonal 2D symmetry. The other materials showed a worm-like mesoporosity.

## 1. Introduction

Periodic mesoporous organosilica materials, at the bulk (PMO) [1,2,3] and nanoscale (PMO NPs) level, are fundamentally unique because they have several advantages, such as robust porous organic-inorganic framework [4], porous channels [5], tunable pore size organization [6,7], biocompatibility [8], and the highest organic content (nano)materials [9]. Moreover, the functionalization of the inner and outer surface allows the modulation of the properties and enable dispersibility in aqueous or organic solvents [10]. The hydrophilicity/hydrophobicity of the pores is also adjustable [11,12]. The PMO (and PMO NPs) can be degraded when specific functional groups sensitive to redox, acid-base, biochemical, or photochemical reactions are present in the structure of the organic framework [13,14,15]. 

The post-modification of the organic fragment is also possible by classical organic chemistry [16,17]. Especially in nanomedicine, properties of PMO NPs such as low hemolytic behavior [18], increased biocompatibility, higher loading capacities, physico-chemical adjustability of the pores [19] and designed biodegradability [20], are particularly important when compared to mesoporous silica nanoparticles.

PMO and PMO NPs are synthesized from organo-bridged alkoxysilanes by the sol-gel process in the presence of surfactants that act as templates. The incorporation of organic moieties as bridging components, directly and specifically into the pore walls, by using disilylated organosilica precursors, enable the preparation of a new generation of organosilica (nano)materials that combines the advantages of organic and inorganic units while overcoming their intrinsic disadvantages [6,20,21,22,23]. If there is only one bridged organosilica precursor, pure PMO NPs can be formed. When two bridged organosilica precursors with different functional groups are used, mixed PMO NPs are obtained with two different functionalities in the framework wall. 

Specifically, the synthesis of PMO NPs implies the careful control of a range of conditions, such as the reaction temperature, reaction time, pH, solvent, precursors, optimized reactants ratio, catalyst, mixing method and stirring speed. The experimental conditions control the template-silica interactions, the silica condensation rate, the assembly kinetics and, thus, the nucleation and growth rates. Control over the nanostructure, morphological uniformity, size distribution, porosity and compositions of PMO NPs is quite challenging. Interestingly, different types of PMO NPs can be obtained through templating strategies such as spherical nanoparticles, nanorods, nanofibers, and multipodal NPs [9,23].

Although a broad array of PMO materials have been reported, achieving the nanoscale for such materials is still challenging. The first PMO NPs with a hollow spherical morphology (HPMO NPs) were prepared from 1,2-bis(trimethoxysilyl)ethane by using the FC-4 fluorocarbon surfactant [C_3_F_7_O(CFCF_3_CF_2_O)_2_CFCF_3_CONH(CH_2_)_3_N^+^(C_2_H_5_)_2_CH_3_, I^−^] and CTAB cationic surfactant as co-structure directing agents [24]. In addition, by changing the ratio of FC-4 to CTAB, the shell thickness of HPMO NPs can be adjusted, and non-hollow PMO microspheres can be obtained by using only CTAB surfactant. The procedure followed to obtain phenylene-bridged PMO nanospheres was based on the use of two templates, namely, poly(ethylene oxide)-poly(DL-lactic acid-co-glycolic acid)-poly(ethylene oxide) triblock copolymer and the FC-4 surfactant [25]. Interestingly, the authors found that these two surfactants were immiscible, the core being templated by the polymer based on poly-lactic acid and the shell by FC-4 [25]. When Pluronic P123 block copolymer alone was used as template (without FC-4), hollow silica nanospheres were obtained instead of the expected PMO material [26]. Later, a variety of PMO nanospheres with different organic bridged linkers, such as methylene, ethylene, ethynylene and phenylene was reported [18,26,27]. In 2005, Lu and colleagues were the first to use Pluronic P123 triblock copolymer as a structure-directing agent to control the synthesis of ethylene bridged PMO nanorods [28]. Jaroniec and colleagues then described the use of both 1,2-bis (triethoxysilyl)ethane (BTEE) and 1,4-bis (triethoxysilyl)benzene (BTEB) as organosilica precursors, and Pluronic P123 poly(ethylene oxide)-poly(propylene oxide)-poly(ethylene oxide) (PEO-PPO-PEO) as triblock copolymer template to control the synthesis of ethylene-phenylene wide microrods with coil-like morphology [29]. In 2009, Mohanty and Landskron used the trisilylated octaethoxy-1,3,5-trisilapentane as precursor with P123 surfactant, and PMO nanorods and nanofibers were obtained with an adjustable aspect ratio from 2:1 to 20:1 by varying the concentration of the precursor [30,31]. In 2011, methylene-, ethenylene-, and phenylene-bridged PMO helical fibers were prepared using CTAB and (*S*)-β-citronellol as templating agents [32]. The sizes and shapes could be adjusted to obtain nanorods depending on the precursor used. In some research works, CTAB was used as the only template to prepare bridged PMO nanorods [7,11]. Interestingly, Khashab’s group reported the first controlled syntheses of *para*-phenylene-bridged PMO NPs with different morphologies, from nanospheres to nanorods and nanowires, with CTAB as a structure-directing agent by adjusting the organic co-solvents [33]. Multipodal PMO NPs with phenylene-bridged cores and ethenylene-bridged pods were designed by Croissant et al., which were prepared in a CTAB-templated aqueous medium with sodium hydroxide as catalyst through a one-pot two-step method [34]. While the sole condensation of the 1,4-bis(triethoxysilyl)benzene or 1,2-bis-(triethoxysilyl)ethylene precursor afforded phenylene- and ethenylene-bridged PMO nanospheres and nanorods, respectively, the addition of the ethenylene precursor into a solution of freshly prepared phenylene-bridged NPs gave rise to multipodal nano-objects. Interesting reviews on the synthetic strategies for the preparation of PMO nanomaterials and their applications in nanomedicine and catalysis have been published [20,23,35,36,37]. Furthermore, the glutathione-triggered degradability of PMO and related materials by incorporating disulfide bridges in the structure has been comprehensively reviewed [14]. 

The synthesis of PMO NPs with large and flexible organic bridging groups within the mesoporous wall remains a scientific challenge and the development of new mixed periodic mesoporous organosilica nanoparticles is very relevant, especially for potential new applications in nanomedicine.

**Figure 1:**
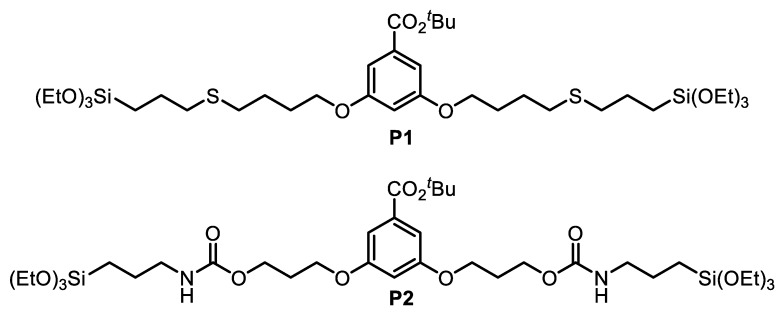
Proposed disilylated *tert*-butyl 3,5-dialkoxybenzoates **P1** and **P2** for the preparation of mixed **PMO NPs.**

Herein, we describe the preparation and full characterization of novel mixed PMO NPs which have been obtained by sol-gel co-condensation of *E*-1,2-bis(triethoxysilyl)ethylene (**BTSE** or **E**) [38] with disilylated *tert*-butyl 3,5-dialkoxybenzoates bearing either sulfide (precursor **P1**) or carbamate (precursor **P2**) functionalities in the linker (Figure 1).

## 2. Materials and Methods 

### 2.1. General Remarks

The ^13^C-CP-MAS solid state NMR spectra (Les Ullis, France), the BET surface areas (Mérignac, France), Dynamic Light Scattering (DLS, Pessac, France) and Zeta potential values (Orsay, France) were obtained from equipments of *University of Montpellier* [39]. The ^1^H- and ^13^C-NMR spectroscopy (Bruker Biospin, Rheinstetten, Germany), Mass-Spectrometry (MS, Bruker Biosciences Española, Madrid, Spain), elemental analysis, Infra-Red spectroscopy (IR, Bruker Biosciences Española, Madrid, Spain), Powder X-Ray diffraction (*P*-XRD, Almelo, Netherlands) and Transmission Electron Microscopy (TEM, JEOL Ltd., Akishima, Tokyo, Japan) were performed at the *Universitat Autònoma de Barcelona* (UAB) [39]. 

When necessary, experiments were carried out with Schlenk techniques under standard high vacuum. CTAB, NH_4_NO_3_, sodium hydroxide, potassium bromide and the required reagents for the preparation of **P1** and **P2** were purchased from Sigma-Aldrich (Madrid, Spain). Ethanol and acetone were purchased from Fisher Chemicals (Madrid, Spain) and hydrochloric acid from VWR PROLABO (Barcelona, Spain). **E PMO NPs** were prepared as described [11]. 

### 2.2. Synthesis of ***2***

In a 250 mL Schlenk flask under nitrogen atmosphere, but-3-en-1-ol **1** (8.0 mL, 0.843 g/mL, 93.50 mmol) was added, dissolved in dry dichloromethane (100 mL) and dry triethylamine (5.0 mL, 0.73 g/ mL, 117 mmol). Then, the mixture was stirred with cooling (ice bath) over a period of 15 min. After this time, methanesulfonyl chloride (15.0 mL, 1.48 g/mL, 193.80 mmol) was added and the reaction mixture was stirred overnight at room temperature under argon atmosphere. Then, 100 mL of fresh dichloromethane were added to the crude and it was washed first with HCl 5 M (50 mL × 2) and then with saturated aqueous solution of Na_2_CO_3_ (50 mL × 2) and brine (50 mL × 2). The organic fraction was dried using anhydrous sodium sulfate and the solvent evaporated under vacuum to afford the final product **2** as yellow liquid (13.80 g, 99% yield) [40]. ^1^H NMR (360 MHz, CDCl_3_) *δ* (ppm): 5.84–5.73 (m, 1H), 5.21–5.14 (m, 2H), 4.27 (t, *J* = 7.2 Hz, 2H), 3.01 (s, 3H), 2.54–2.48 (m, 2H).

### 2.3. Synthesis of ***4***

To the stirred mixture of 3,5-dihydroxybenzaldehyde **3** (1.38 g, 10.0 mmol) and cesium carbonate (13.03 g, 40.0 mmol) in anhydrous DMF (80 mL) an excess of but-3-en-1-yl methanesulfonate **2** (4.50 g, 30.0 mmol) was added. The reaction was stirred at 40 °C under argon atmosphere until 3,5-dihydroxybenzaldehyde was fully consumed (TLC monitoring, 48 h). Then, water was added (100 mL) and the product was extracted using hexane (200 mL × 3). The organic phase was washed with 1M NaOH, dried with anhydrous sodium sulfate and the solvent was removed under vacuum to afford the final product **4** as yellow liquid (1.93 g, 78% yield) [41]. ^1^H NMR (360 MHz, CDCl_3_) *δ* (ppm): 9.89 (s, 1H), 7.00 (br s, 2H), 6.71 (br s, 1H), 5.93–5.84 (m, 2H), 5.20-5.11 (m, 4H), 4.05 (t, *J* = 7.2 Hz, 4H), 2.58–2.55 (m, 4H); ^13^C NMR (91 MHz, CDCl_3_) *δ* (ppm): 192.0, 160.5, 138.3, 134.1, 117.3, 108.1, 107.8, 67.6, 33.5. IR (film): 3078.9, 2980.0, 2876.7, 1697.7, 1592.4, 1452.6, 1295.6, 1296.0, 1165.9, 1065.9, 1065.1, 843.9, 676.0 cm^−1^. GC-MS (EI) m/z: 246.2 [M^+^], 192.1, 163.1, 150.1, 138.0, 121.1, 110.0, 91.1, 75.0, 65.1, 55.1; HRMS (ESI) m/z [M + Na]^+^ calcd for C_15_H_18_O_3_Na: 269.1148, found: 269.1141.

### 2.4. Synthesis of ***5***

To a stirred solution of 3,5-bis(but-3-en-1-yloxy)benzaldehyde **4** (1.97 g, 8.0 mmol) in acetonitrile (12.0 mL) was added a solution of NaH_2_PO_4_·2H_2_O (374.4 mg, 2.4 mmol) in water (5.0 mL) and 30% H_2_O_2_ (4.0 mL, 9.97 mol/L, 40.0 mmol). Finally, a solution of 80% purity NaClO_2_ (1.27 g, 11.2 mmol) in water (10.0 mL) was added dropwise during 2 h to the stirred mixture at room temperature. O_2_ evolved from the solution was monitored until the end of the reaction with a bubbler connected to the flask. A small amount of Na_2_SO_3_ was added to destroy the excess of oxidants. The mixture was acidified with 10% aqueous HCl, and the product was extracted using ethyl acetate (60 mL × 3). The organic phase was dried with anhydrous sodium sulfate and the solvent was removed under vacuum to afford a residue which was purified by a flash column chromatography through silica gel eluting with hexane/EtOAc = 2:1, obtaining the product **5** as a colorless solid (1.78 g, 85% yield) [42]. ^1^H NMR (360 MHz, CDCl_3_) *δ* (ppm): 7.25 (d, *J* = 3.6 Hz, 2H), 6.70 (br s, 1H), 5.94–5.85 (m, 2H), 5.21–5.11 (m, 4H), 4.05 (t, *J* = 7.2 Hz, 4H), 2.58–2.53 (m, 4H); ^13^C NMR (91 MHz, CDCl_3_) *δ* (ppm): 171.7, 160.0, 134.2, 130.9, 117.2, 108.4, 107.6, 67.5, 33.5. 

### 2.5. Synthesis of ***6***

In a 10 mL round bottom flask, 3,5-bis(but-3-en-1-yloxy)benzoic acid **5** (1.31 g, 5.0 mmol), di-*tert*-butyl dicarbonate (2.18 g, 10.0 mmol) and 4-dimethylaminopyridine (183.3 mg, 1.5 mmol) were dissolved in *tert*-butanol (40 mL). The reaction mixture was stirred at room temperature under argon atmosphere until 3,5-bis(but-3-en-1-yloxy)benzoic acid **5** was fully consumed (TLC monitoring, 48 h). Then, the solvent was removed under reduced pressure and the residue was purified by flash column chromatography through silica gel eluting with hexane/AcOEt = 15:1, obtaining the pure product **6** as colorless oil (1.38 g, 87% yield) [43]. ^1^H NMR (360 MHz, CDCl_3_) *δ* (ppm): 7.13 (d, *J* = 3.6 Hz, 2H), 6.62 (t, *J* = 3.6 Hz, 1H), 5.94–5.84 (m, 2H), 5.20–5.10 (m, 4H), 4.03 (t, *J* = 7.2 Hz, 4H), 2.56–2.53 (m, 4H), 1.58 (s, 9H); ^13^C NMR (91 MHz, CDCl_3_) *δ* (ppm): 165.5, 159.8, 134.3, 133.9, 117.1, 107.8, 105.9, 81.2, 67.4, 33.5, 28.2. IR (film): 3078.7, 2977.7, 1711.7, 1594.3, 1445.8, 1300.5, 1159.9, 1049.1, 915.6, 767.2 cm^−1^. GC-MS (EI) m/z: 318.2 [M]^+^, 262.1, 245.2, 208.1, 190.1, 166.1, 154.0, 136.0, 55.1; HRMS (ESI) m/z [M + Na]^+^ calcd for C_19_H_26_O_4_Na: 341.1723, found: 341.1707.

### 2.6. Synthesis of ***P1***

In a 50 mL Schlenk tube under nitrogen, *tert*-butyl 3,5-bis(but-3-en-1-yloxy)benzoate **6** (954.5 mg, 3.0 mmol) and 2,2-dimethoxy-1,2-diphenylethanone (DMPA) (153.8 mg, 0.6 mmol) were dissolved in anhydrous THF (12.0 mL). Then (3-mercaptopropyl)triethoxysilane (1.5 g, 6.3 mmol) was added and the stirred mixture was irradiated with a UV lamp at 365 nm under argon atmosphere until *tert*-butyl 3,5-bis(but-3-en-1-yloxy)benzoate **6** was fully consumed (TLC monitoring, 16 h). Then, the solvent was removed under vacuum and the crude product was purified by a flash column chromatography through silica gel eluting with hexane/AcOEt = 10:1, obtaining the pure product **P1** as colorless oil (1.66 g, 70% yield) [44]. ^1^H NMR (360 MHz, CDCl_3_) *δ* (ppm): 7.09 (br s, 2H), 6.58 (br s, 1H), 3.97 (t, *J* = 7.2 Hz, 4H), 3.82 (q, *J* = 7.2 Hz, 12H), 2.56 (apparent q, *J* = 7.2 Hz, 8H), 1.88-1.76 (m, 4H), 1.74–1.70 (m, 8H), 1.57 (s, 9H), 1.21 (t, *J* = 7.2 Hz, 18H), 0.74 (t, *J* = 7.2 Hz, 4H); ^13^C NMR (91 MHz, CDCl_3_) *δ* (ppm): 165.6, 159.9, 133.8, 107.6, 105.7, 81.2, 67.6, 58.4, 35.1, 31.6, 28.4, 28.1, 26.2, 23.2, 18.3, 9.9. IR (film): 2972.2, 2925.1, 1713.7, 1594.6, 1445.3, 1389.1, 1326.1, 1248.8, 1161.9, 1074.2, 956.6, 768.1 cm^−1^. MS (ESI) m/z: 817.4 [M + Na]^+^, 749.4, 647.3, 425.3, 393.3; HRMS (ESI) m/z [M + Na]^+^ calcd for C_37_H_70_O_10_S_2_Si_2_Na: 817.3841, found: 817.3846.

### 2.7. Synthesis of ***9***

To a solution of propane-1,3-diol **8** (7.60 g, 100 mmol) in 200 mL of anhydrous THF was added NaH (4.40 g, 60% dispersion in mineral oil, 110 mmol) portionwise. The mixture was stirred at room temperature for 30 min, then benzyl bromide (18.90 g, 110 mmol) was added dropwise, then tetrabutylammonium iodide (7.40 g, 20 mmol) was added in one portion. The mixture was heated to 60 °C and stirred overnight. After cooling, an equal volume of water was added, and the mixture was extracted with diethyl ether. The combined organic layer was washed with brine, dried over anhydrous Na_2_SO_4_, and filtered. The solvent was evaporated, and the resulting residue was purified by flash column chromatography on silica gel with hexane/EtOAc = 5:1 to afford a yellow liquid (13.80 g, 83% yield) [45]. ^1^H NMR (360 MHz, CDCl_3_) *δ* (ppm): 7.38–7.28 (m, 5H), 4.53 (s, 2H), 3.80 (apparent q, *J* = 7.2 Hz, 2H), 3.67 (t, *J* = 7.2 Hz, 2H), 2.28 (t, *J* = 3.6 Hz, 1H), 1.91–1.84 (m, 2H).

### 2.8. Synthesis of ***10***

To a solution of 3-(benzyloxy)propan-1-ol **9** (9.30 g, 56.0 mmol) in 180 mL of CH_2_Cl_2_ was added PPh_3_ (15.42 g, 58.8 mmol) and imidazole (4.57g, 61.6 mmol), followed by the portionwise addition of iodine (15.63 g, 61.6 mmol) at 0 °C (ice bath). Then the reaction mixture was stirred at rt for 16 h and then quenched with aqueous Na_2_SO_3_ solution. The organic phase was separated, and the aqueous phase was extracted with CH_2_Cl_2_. The combined organic layer was dried with Na_2_SO_4_ and then filtered. The solvent was evaporated, the residue was purified by flash column chromatography through silica gel eluting with hexane, obtaining a colorless liquid (12.1 g, 78% yield) [45]. ^1^H NMR (360 MHz, CDCl_3_) *δ* (ppm): 7.38–7.27 (m, 5H), 4.52 (s, 2H), 3.54 (t, *J* = 7.2 Hz, 2H), 3.31 (t, *J* = 7.2 Hz, 2H), 2.13–2.06 (m, 2H).

### 2.9. Synthesis of ***11***

An excess of [(3-iodopropoxy)methyl]benzene **10** (10.35 g, 37.5 mmol) was added to a stirred solution of 3,5-dihydroxybenzaldehyde **6** (2.07 g, 15 mmol) and cesium carbonate (24.44 g, 75.0 mmol) in anhydrous DMF (120 mL). The reaction was stirred at 40 °C under argon atmosphere until 3,5-dihydroxybenzaldehyde **6** was fully consumed (TLC monitoring, 41 h). Then, water was added (60 mL) and the crude product was extracted with EtOAc (80 mL × 3). The organic phase was washed with water (3 times), dried with anhydrous Na_2_SO_3_ and the solvent was removed under vacuum to afford the crude product as yellow liquid. The pure product was obtained by a flash column chromatography through silica gel eluting with hexane/EtOAc = 5:1, colorless oil (6.07 g, 93% yield) [45]. ^1^H NMR (360 MHz, CDCl_3_) *δ* (ppm): 9.88 (s, 1H), 7.33–7.27 (m, 10H), 7.00 (d, *J* = 3.6 Hz, 2H), 6.69 (t, *J* = 3.6 Hz, 1H), 4.53 (s, 4H), 4.12 (t, *J* = 7.2 Hz, 4H), 3.66 (t, *J* = 7.2 Hz, 4H), 2.13–2.05 (m, 4H); ^13^C NMR (91 MHz, CDCl_3_) *δ* (ppm): 192.1, 160.6, 151.8, 138.3, 128.4, 127.7, 127.6, 108.0, 107.7, 73.1, 66.6, 65.3, 29.6. IR (film): 3029.5, 2932.3, 1696.8, 1591.9, 1452.1, 1295.4, 1164.7, 1094.2, 734.2, 696.7 cm^−1^. ESI-MS m/z: 457.2 [M + Na]^+^, 306.9, 249.9, 181.1, 165.1, 141.1, 91.1; HRMS (ESI) m/z [M + Na]^+^ calcd for C_27_H_30_O_5_Na: 457.1985, found: 457.1984.

### 2.10. Synthesis of ***12***

3,5-Bis[3-(benzyloxy)propoxy]benzaldehyde **11** (3.70 g, 8.5 mmol) was dissolved in acetonitrile (20 mL), then a solution of NaH_2_PO_4_·2H_2_O (358.0 mg, 2.30 mmol) in water (5.0 mL) and 30% H_2_O_2_ (1.1 mL, 9.97 mol/L, 10.97 mmol) was added. Finally, a solution of 80% purity NaClO_2_ (1.36 g, 12.0 mmol) in water (12 mL) was added dropwise during 2 h to the stirred mixture at room temperature. The O_2_ produced from the solution was monitored until the end of the reaction with a bubbler connected to the flask. A little amount of Na_2_SO_3_ was added to destroy the unreacted oxidants. The mixture was acidified with 10% aqueous HCl, and the product was extracted using ethyl acetate (80 mL × 3). The organic phase was dried with anhydrous sodium sulfate and the solvent was removed under vacuum to afford the crude product, which was purified by a flash column chromatography through silica gel eluting with hexane/EtOAc = 1:2, white solid (3.78 g, 99% yield) [42]. ^1^H NMR (360 MHz, CDCl_3_) *δ* (ppm): 7.32–7.24 (m, 12H), 6.67 (br s, 1H), 4.53 (s, 4H), 4.12 (t, *J* = 7.2 Hz, 4H), 3.67 (t, *J* = 7.2 Hz, 4H), 2.18–2.05 (m, 4H); ^13^C NMR (91 MHz, CDCl_3_) *δ* (ppm): 160.1, 152.7, 138.3, 129.8, 128.4, 127.7, 127.6, 108.3, 107.4, 73.1, 66.6, 65.2, 29.6. IR (film): 3651.4, 3024.9, 2861.2, 1686.4, 1593.5, 1445.7, 1393.4, 1297.5, 1169.4, 1091.7 cm^−1^. ESI-MS m/z: 473.2 [M + Na]^+^, 433.2, 283.1, 239.1, 181.1, 122.1; HRMS (ESI): m/z [M + Na]^+^ calcd for C_27_H_30_O_6_Na: 473.1935, found: 473.1937.

### 2.11. Synthesis of ***13***

In a 10-mL round bottom flask, 3,5-bis[3-(benzyloxy)propoxy]benzoic acid **12** (5.40 g, 12.0 mmol), di-*tert*-butyl dicarbonate (5.24 g, 24.0 mmol) and 4-dimethylaminopyridine (439.8 mg, 3.6 mmol) were dissolved in *tert*-butanol (80 mL). The reaction mixture was stirred at 40 °C under argon atmosphere until 3,5-bis[3-(benzyloxy)propoxy]benzoic acid **12** was fully consumed (TLC monitoring, 48 h). Then, the solvent was removed under vacuum and the crude product was purified by flash column chromatography through silica gel eluting with hexane/AcOEt = 10:1, obtaining the pure product **13** as colorless oil (4.25 g, 70% yield) [43]. ^1^H NMR (360 MHz, CDCl_3_) *δ* (ppm): 7.33–7.12 (m, 10H), 7.13 (d, *J* = 3.6 Hz, 2H), 6.60 (t, *J* = 3.6 Hz, 1H), 4.52 (s, 4H), 4.09 (t, *J* = 7.2 Hz, 4H), 3.65 (t, *J* = 7.2 Hz, 4H), 2.10–2.04 (m, 4H), 1.58 (s, 9H); ^13^C NMR (91 MHz, CDCl_3_) *δ* (ppm): 165.6, 159.6, 138.4, 133.9, 128.4, 127.65, 127.60, 107.8, 105.8, 81.1, 73.1, 66.7, 65.1, 29.7, 28.2. IR (film): 3651.4, 2930.3, 2860.4, 1710.1, 1593.9, 1445.8, 1300.6, 1160.7, 1096.4, 731.9, 696.7 cm^−1^. ESI-MS m/z: 529.3 [M + Na]^+^, 451.3, 433.2, 283.1, 265.1, 181.1, 122.1; HRMS (ESI): m/z [M + Na]^+^ calcd for C_31_H_38_O_6_Na: 529.2561, found: 529.2555.

### 2.12. Synthesis of ***14***

A mixture of *tert*-butyl 3,5-bis[3-(benzyloxy)propoxy]benzoate **13** (1.40 g, 2.77 mmol) and preequilibrated 10% Pd/C (0.308 g, 0.029 mmol Pd) in absolute ethanol (20 mL) was hydrogenated at room temperature and atmospheric pressure. When *tert*-butyl 3,5-bis[3-(benzyloxy)propoxy]benzoate **13** was fully consumed (TLC monitoring, 17 h), the reaction mixture was filtered through celite^®^ 545, the filtrate was evaporated to give the crude product. The final pure product was obtained by flash column chromatography (silica gel eluting with hexane/EtOAc = 1:2), white solid (858 mg, 95% yield) [46]. ^1^H NMR (360 MHz, CDCl_3_) *δ* (ppm): 7.14 (d, *J* = 3.6 Hz, 2H), 6.62 (t, *J* = 3.6 Hz, 1H), 4.16–4.12 (m, 4H), 3.85 (t, *J* = 7.2 Hz, 4H), 2.07–2.01 (m, 4H), 1.58 (s, 9H); ^13^C NMR (91 MHz, CDCl_3_) *δ* (ppm): 165.4, 159.7, 134.0, 107.8, 105.8, 81.3, 65.9, 60.3, 31.9, 28.1. IR (film): 3274.5, 2967.9, 2870.6, 1705.7, 1595.1, 1447.7, 1349.7, 1299.2, 1169.9, 1058.6, 847.4, 767.4 cm^−1^. ESI-MS m/z: 349.2 [M + Na]^+^, 293.1, 271.1, 253.1, 195.1, 122.1; HRMS (ESI): m/z [M + Na]^+^ calcd for C_17_H_26_O_6_Na: 349.1622, found: 349.1618.

### 2.13. Synthesis of ***P2***

In a 100-mL Schlenk tube under nitrogen, *tert*-butyl 3,5-bis(3-hydroxypropoxy)benzoate **14** (1.96 g, 6.0 mmol) and 3-(isocyanatopropyl)triethoxysilane **15** (7.42 g, 7.42 mL, 1.0 g/mL, 30.0 mmol) were dissolved in dry THF (20 mL). The mixture was stirred under reflux and argon atmosphere. After 17 h, the starting material was fully consumed (TLC monitoring). Then, the solvent was removed under reduced pressure and the residue was purified by flash column chromatography through silica gel eluting with hexane/EtOAc = 2:1 to obtain the pure product **P2** as colorless oil (4.90 g, 100% yield). ^1^H NMR (360 MHz, CDCl_3_) *δ* (ppm): 7.12 (broad s, 2H), 6.59 (t, *J* = 3.6 Hz, 1H), 4.99 (br s, 2H), 4.22 (t, *J* = 7.2 Hz, 4H), 4.05 (t, *J* = 7.2 Hz, 4H), 3.81 (q, *J* = 7.2 Hz, 12H), 3.18 (q, *J* = 7.2 Hz, 4H), 2.09-2.04 (m, 4H), 1.72–1.61 (m, 4H), 1.57 (s, 9H), 1.21 (t, *J* = 7.2 Hz, 18H), 0.62 (t, *J* = 7.2 Hz, 4H); ^13^C NMR (91 MHz, CDCl_3_) *δ* (ppm): 165.5, 159.7, 156.5, 133.8, 107.7, 105.8, 81.2, 64.6, 61.3, 58.4, 43.4, 28.9, 28.1, 23.3, 18.3, 7.6. IR (film): 3343.6, 2972.7, 2927.5, 1708.6, 1595.9, 15527.2, 1242.3, 1163.0, 1071.9, 953.2, 767.1 cm^−1^. ESI-MS m/z: 843.1 [M + Na]^+^, 775.4, 729.3, 673.3; HRMS (ESI): m/z [M + Na]^+^ calcd for C_37_H_68_N_2_O_14_Si_2_Na: 843.4101, found: 843.4108.

### 2.14. General Procedure for the Preparation of ***E-Pn 90/10*** or ***75/25 PMO NPs***

A solution of CTAB (250 mg, 0.686 mmol) in Mili-Q water (120 mL) was placed in a 250 mL round bottom flask, then 2 M NaOH (875 µL) was added (1.75 mmol of NaOH). The mixture was stirred at 1000 rpm at 80 °C for 50 min. After that, the stirring speed was increased to 1400 rpm and a mixture of 100% *E*-BTSE with **Pn** (for **90/10**: 2.00 mmol *E*-BTSE and 0.2 mmol **Pn**; for **75/25**: 1.65 mmol *E*-BTSE and 0.55 mmol **Pn**) was added rapidly under stirring. The condensation process was conducted for another 120 min at 80 °C. Afterwards, the suspension was cooled to room temperature and the nanoparticles were collected by centrifugation (13500 rpm for 45 min). The samples were then extracted with a solution of NH_4_NO_3_ (6 g/L in 96% EtOH) for three times and washed successively with 96% ethanol, Mili-Q water, 96% ethanol. The **E-Pn PMO NPs** were obtained as white solids [39]. **E-P1 90/10 PMO NPs**: ^13^C-CP-MAS NMR (75 MHz) *δ* (ppm): 165, 160, 146, 134, 107, 82, 68, 58, 33, 28, 24, 17, 13. IR *ν* (ATR) (cm^−1^): 3311.8, 2975.4, 1692.3, 1596.5, 1449.9, 1187.3, 1033.1, 923.2, 788.5. BET: S_BET_ = 973 m^2^g^−1^, V_pore_ = 0.66 cm^3^g^−1^, ∅_pore_ = 2.73 nm. TGA (air, 5 °C /min, 20–1000 °C) residual mass 77%. Zeta Potential: ζ = −30.9 mV, pH = 6.80. DLS: 1122 nm. **E-P2 90/10 PMO NPs**: ^13^C-CP-MAS NMR (75 MHz) *δ* (ppm): 165, 160, 146, 134, 109, 81, 63, 58, 44, 28, 24, 17, 11. IR *ν* (ATR) (cm^−1^): 3316.2, 1695.5, 1448.5, 1187.9, 1035.2, 923.1, 789.2. BET: S_BET_ = 696 m^2^g^−1^, V_pore_ = 0.37 cm^3^g^−1^, ∅_pore_ = 2.13 nm. TGA (air, 5 °C/min, 20–1000 °C) residual mass 75%. Zeta Potential: ζ = −38.2 mV, pH = 6.72. DLS: 694 nm. **E-P1 75/25 PMO NPs**: ^13^C-CP-MAS NMR (75 MHz) *δ* (ppm): 165, 160, 146, 134, 107, 81, 68, 58, 34, 29, 18, 13. IR *ν* (ATR) (cm^−1^): 3359.1, 1709.4, 1594.2, 1447.0, 1035.6, 924.5, 790.3. BET: S_BET_ = 259 m^2^g^−1^, V_pore_ = 0.17 cm^3^g^−1^, ∅_pore_ = 2.59 nm. TGA (air, 5 °C/min, 20–1000 °C) residual mass 67%. Zeta Potential: ζ = −25.6 mV, pH = 9.07. DLS: 742 nm. **E-P2 75/25 PMO NPs**: ^13^C-CP-MAS NMR (75 MHz) *δ* (ppm): 166, 160, 158, 146, 134, 111, 105, 81, 64, 58, 44, 28, 24, 18, 11. IR *ν* (ATR) (cm^−1^): 3340.1, 1692.2, 1447.3, 1188.5, 1035.8, 922.5, 791.1. BET: S_BET_ = 329 m^2^g^−1^, V_pore_ = 0.26 cm^3^g^−1^, ∅_pore_ = 2.74 nm. **TGA** (air, 5 ^o^C/min, 20–1000 °C) residual mass 63%. Zeta Potential: ζ = −42.3 mV, pH = 6.85. DLS: 562 nm.

## 3. Results and Discussion

### 3.1. Synthesis of the Disilylated Precursors ***P1*** and ***P2***

**Scheme 1:**
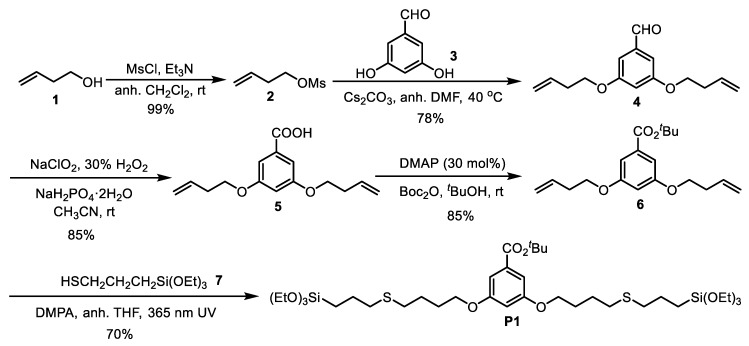
Synthesis of precursor **P1.**

The synthesis of **P1** is depicted in Scheme 1. We first converted commercial 3-buten-1-ol **1** into but-3-en-1-yl methanesulfonate **2 [40]**. The dialkylation of commercial 3,5-dihydroxybenzaldehyde, **3**, with the mesylate **2**, in the presence of cesium carbonate in DMF at 40 °C, afforded **4** in 78% isolated yield after chromatographic purification [41]. The aldehyde **4** was oxidized to the carboxylic acid **5** by using NaClO_2_, 30% H_2_O_2_ and NaH_2_PO_4_ in aqueous acetonitrile at room temperature [42]. Subsequent treatment of **5** with Boc_2_O and 4-dimethylaminopyridine (DMAP) in *tert*-butanol at room temperature provided the ester **6** in 85% isolated yield [43]. Finally, the precursor **P1** was obtained in 70% isolated yield by the versatile thiol-alkene click reaction between dialkene **6** and (3-mercaptopropyl)triethoxysilane **7** under irradiation at 365 nm using 2,2-dimethoxy-1,2-diphenylethanone (DMPA) as initiator [44].

**Scheme 2:**
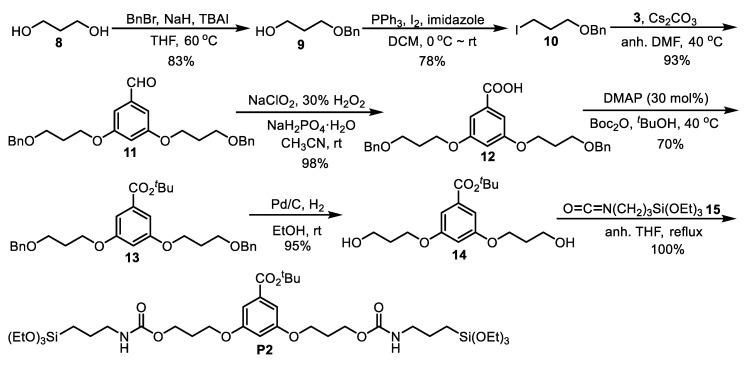
Synthesis of precursor **P2.**

In Scheme 2**,** we summarize the preparation of precursor **P2**. We first synthesized [(3-iodopropoxy)methyl)]benzene **10** (65% overall yield) from the commercially available 1,3-propanediol **8** through the monobenzylated intermediate **9 [45]**. Then, commercial 3,5-dihydroxybenzaldehyde **3** was dialkylated with the iodide **10** to obtain the aldehyde **11**. The next steps were oxidation to the carboxylic acid **12**, formation of *tert*-butyl ester **13**, followed by deprotection of benzylic ethers, leading to the obtention of diol **14** in high yield [46]. Subsequent treatment of this compound with the silylated isocyanate **15** provided the disilylated carbamate precursor **P2** in quantitative yield.

### 3.2. Preparation and Characterization of ***E-Pn 90/10 PMO NPs*** and ***E-Pn 75/25 PMO NPs***

**Scheme 3:**
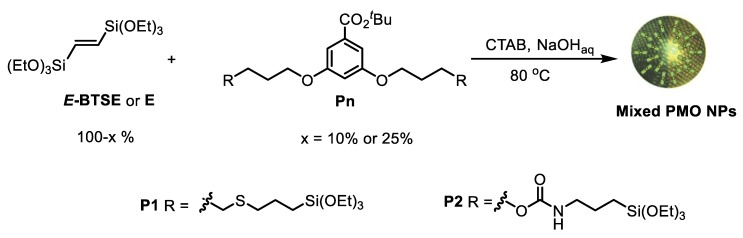
Preparation of E-Pn PMO NPs.

With those precursors **P1** and **P2** in hand, a series of **E-Pn PMO NPs** were prepared by mixing the corresponding disilylated precursor and (*E*)-BTSE (named **E**) as major reagent [38] The syntheses were performed in Mili-Q water with CTAB as template under basic catalysis (NaOH). The micellar solution was prepared at 80 °C for 50 min with a stirring speed adjusted at 1000 rpm. Then, the stirring speed was enhanced to 1400 rpm, a mixture of (*E*)-BTSE (or **E**) and different amounts of the corresponding disilylated precursor **Pn** was quickly added and the mixture was left to react for 120 min at 80 °C [39]. **E-Pn PMO NPs** were collected by centrifugation. The template was then removed by washing with a solution of NH_4_NO_3_ (6 g/L in 96% EtOH). Herein, two different ratios of **E/Pn** were prepared, namely 90/10 and 75/25, in order to investigate the influence on the size, morphology and textural properties of the resulting nanomaterials (Scheme 3).

All the mixed PMO NPs were characterized by transmission electron microscopy (TEM), nitrogen-sorption measurements (BET), dynamic light scattering (DLS), zeta-potential, thermogravimetric analysis (TGA),^13^C CP MAS solid state NMR, Fourier-transform infrared spectroscopy (FTIR) and small-angle X-ray diffraction (*p*-XRD). Table 1 shows the physical data of these new materials.

**Figure 2:**
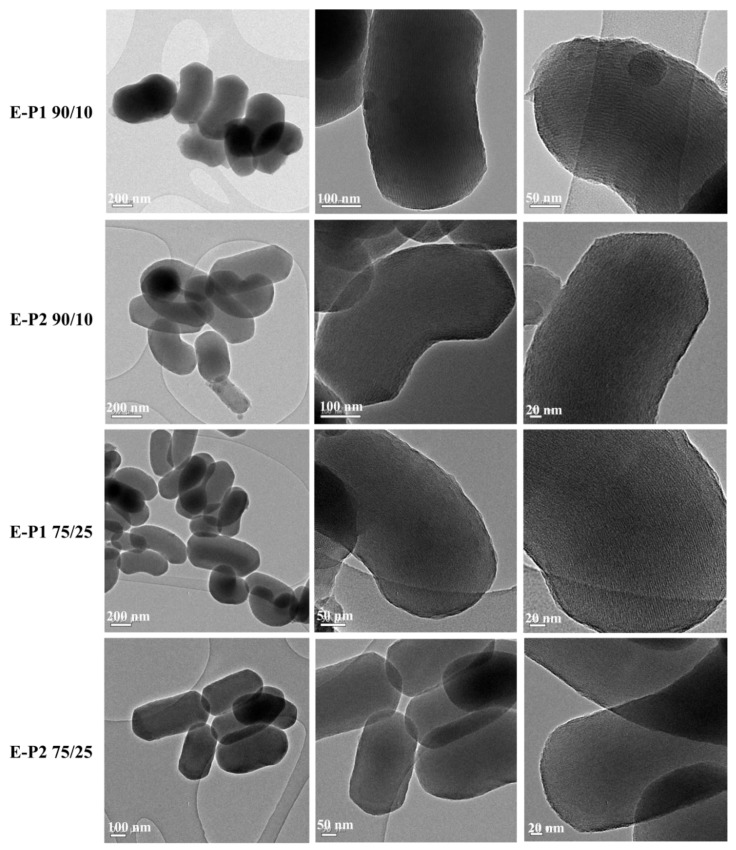
TEM images of E-Pn PMO NPs.

The morphologies of **E-Pn PMO NPs** were first investigated by TEM (Figure 2). All the nanomaterials were obtained as rodlike-shape nanoparticles, similarly to the parent pure **E PMO NPs [11]**, with the length of nanorods ranging from 400 to 600 nm. The hydrodynamic diameters were determined by DLS and the obtained values (from 560 to 1100 nm, Table 1) are consistent with the sizes found by TEM. The zeta potentials (Table 1) were determined in water and gave negative values due to the presence of deprotonated silanol groups at the surface of the nanorods. The high values are indicative of stability.

**Figure 3:**
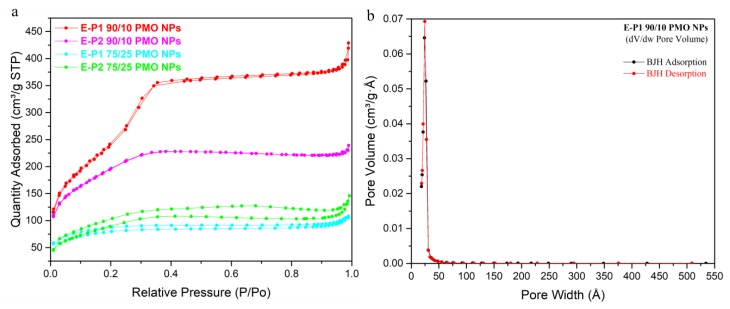
(**a**) N_2_-sorption isotherms of **E-Pn PMO NPs**; (**b**) BJH dV/dw Pore Volume of **E-P1 90/10 PMO NPs.**

The porosity of the nanomaterials was proved by N_2_-sorption experiments (Figure 3). Note that whereas the S_BET_ for materials formed with 25% of **Pn** were found between 259 and 329 m^2^g^−1^, **E-Pn 90/10 PMO NPs** presented high specific surface areas ranging from 696 to 973 m^2^g^−1^ (Table 1), comparable to or even higher than pure **E PMO** nanorods (already synthesized) [11] which showed a specific surface area of 800 m^2^g^−1^. **E-P1 90/10 PMO NPs** presented a type IV isotherm with a small hysteresis in agreement with a mesoporous structure of high specific surface area and pore size of 2.7 nm. **E-P2 90/10 PMO NPs** showed an isotherm between type I and IV. The microporosity is present with a pore size of 2.1 nm and a lower specific surface area than **E-P1 90/10 PMO NPs** of 696 m^2^g^−1^.

**Figure 4:**
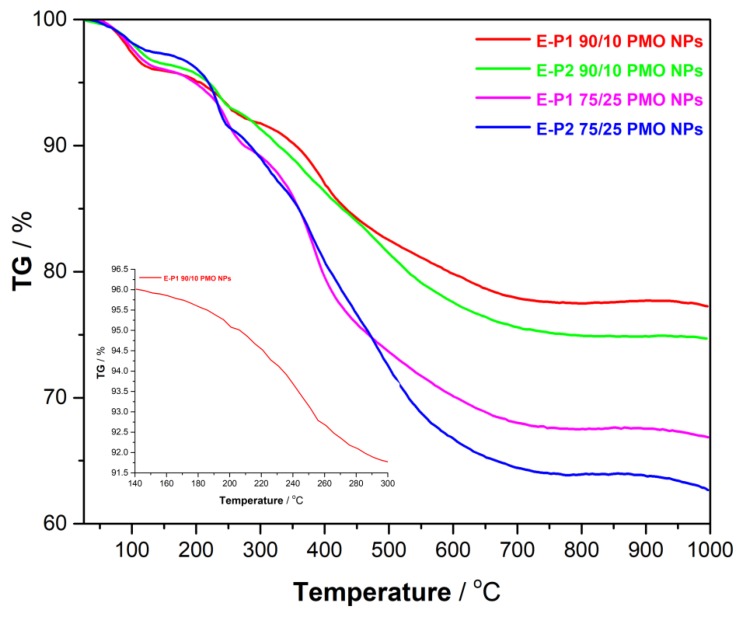
TGA of E-Pn PMO NPs.

In the TGA analyses (Figure 4), the loss of mass corresponding to adsorbed water (up to 120 °C) was observed first. The loss of the *tert*-butoxycarbonyl group took place from 150 °C to 300 °C. Thus, this group was found to be very stable in the materials in solid dry state. Then, at higher temperatures, the decomposition of the remaining organic part of the materials occurred.

**Figure 5:**
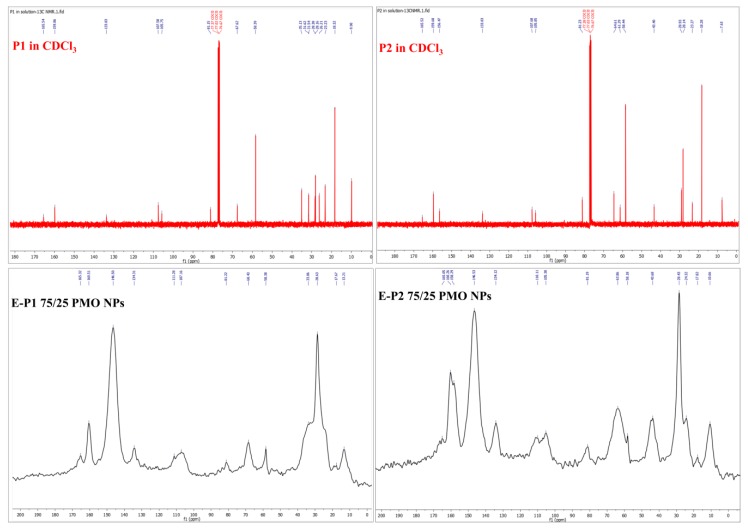
^13^C NMR of **Pn** in CDCl_3_ (above) and ^13^C CP MAS NMR of **E-Pn 75/25 PMO NPs** (below).

^13^C CP MAS solid-state NMR of these new nanomaterials were also recorded. The superposition of the ^13^C NMR spectra of **Pn** in solution and of **E-Pn PMO NPs** in solid state showed a good match, suggesting thereby that the integrity of the organic moiety of the precursor **Pn** had been preserved (Figure 5). For **E-P1 PMO NPs**, the characteristic signals (in ppm) appear at about 165 (C=O of ester), 81 (Cq of *t*Bu), 29 (CH_3_ of *t*Bu), 13 (CH_2_–Si), together with the carbons of the aromatic ring and the methylenic carbons of the linker. A similar pattern is observed for **E-P2 PMO NPs**, with additional signal at about 158 (C=O of carbamate). Moreover, the intense signal at about 146 ppm in both spectra is due to the olefinic carbons of *E*-BTSE.

**Figure 6:**
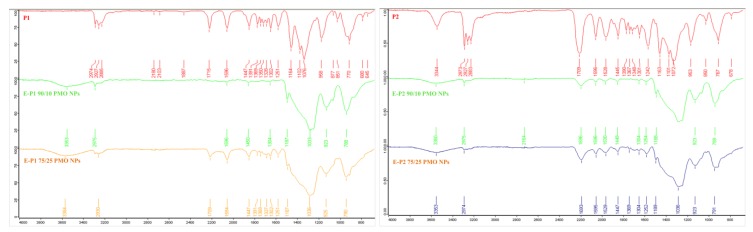
FTIR of Pn and E-Pn PMO NPs.

FTIR spectra of precursors **P1** and **P2** (Figure 6) show the presence of *tert*-butyl group (2974/2973 cm^−1^, υ CH_3_), carbonyl group (1713 cm^−1^, υ C=O of ester in **P1**; broad signal at 1709 cm^−1^ in **P2** for overlapped C=O from the ester and carbamate) and silicon-oxygen bond (υ Si–O at 1076/1072 cm^−1^). The corresponding signals are also visible in **E-Pn 75/25 PMO NPs** (Figure 6), with a shift for the carbonyl group to 1709/1693 cm^−1^. The absorption of C=O in the case of **E-P1 90/10 PMO NPs** is barely visible due to the high dilution of the precursor. The intense and broad band at 1200–1000 cm^−1^ (υ Si–O–Si) is characteristic of a well condensed material.

**Figure 7:**
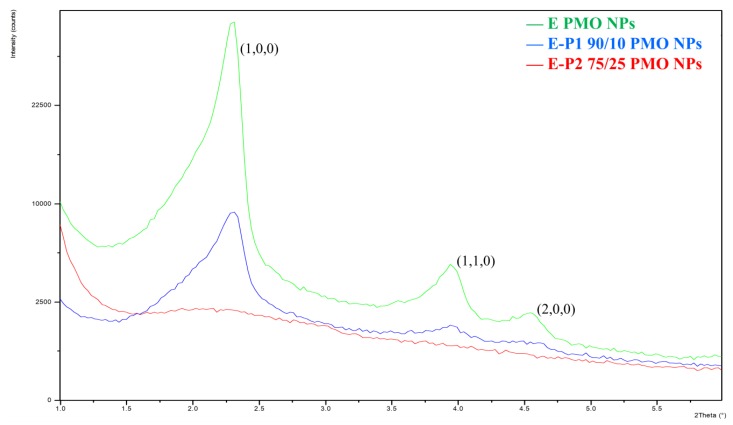
Small angle XRD diagram of **E PMO NPs** (green line), **E-P1 90/10** (blue line), **E-P1 75/25** (red line).

The amall angle X-ray diffraction (*p*-XRD) analyses show an organized porosity for **E-P1 90/10 PMO NPs** typical for a hexagonal 2D symmetry with a sharp Bragg peak [(1,0,0)] and the two first harmonics [(1,1,0) and (2,0,0)]. The other mixed **E-Pn PMO NPs** are disordered, with a broad non-structured band typical of worm-like mesoporosity (Figure 7).

As the synthesis of PMO NPs with large and flexible organic bridging groups within the mesoporous wall is still a challenge, the development of these new mixed periodic mesoporous organosilica nanoparticles is growing. The subsequent hydrolysis of the *tert*-butyl ester groups in those materials would give rise to mixed PMO NPs bearing acidic and coordinating carboxyl groups, which are very relevant for potential applications in nanomedicine for pH-triggered drug delivery [47,48] or in catalysis [49].

## 4. Conclusions

In summary, novel mixed **E-Pn PMO NPs** have been prepared by sol-gel co-condensation of *E*-1,2-bis(triethoxysilyl)ethylene ((*E*)-**BTSE** or **E**) with previously synthesized disilylated *tert*-butyl 3,5-dialkoxybenzoates bearing either sulfide (precursor **P1**) or carbamate (precursor **P2**) functionalities in the linker. Two different ratios of **E/Pn** were examined, namely 90/10 and 75/25, in order to investigate the influence on the size, morphology and textural properties of the resulting nanomaterials. The syntheses were performed in Mili-Q water with CTAB as micellar template under basic catalysis (NaOH) at 80 °C under magnetic stirring.

The mixed **E-Pn PMO NPs** were characterized by TEM, DLS, zeta-potential, nitrogen-sorption measurements (BET), TGA, FTIR, ^13^C CP MAS solid state NMR and *p*-XRD. All the nanomaterials were obtained as mesoporous rodlike-shape nanoparticles, similarly to the parent pure **E PMO NPs**, the length of nanorods ranging from 400–600 nm as demonstrated by TEM. The integrity of the organic skeleton of the corresponding precursor **Pn** has been preserved in the nanomaterials as shown by FTIR and ^13^C CP MAS solid state NMR. Remarkably, whereas the specific surface for **E-Pn 75/25 PMO NPs** were found between 260 and 330 m^2^g^−1^, **E-Pn 90/10 PMO NPs** presented high specific surface areas ranging from 700 to 970 m^2^g^−1^, comparable or even higher than pure **E PMO** nanorods. Moreover, XRD analyses showed an organized porosity for **E-P1 90/10 PMO NPs** typical of hexagonal 2D symmetry. The other materials were disordered with a broad non-structured band that indicated worm-like mesoporosity.

Mixed PMO NPs bearing acidic and coordinating carboxyl groups resulting from the ester hydrolysis would be very relevant for potential applications in catalysis or in nanomedicine for pH-triggered drug delivery.

## Figures and Tables

**Table 1:** Some data of E-Pn PMO NPs.

Material	N_2_-Sorption Measurements			Particles Size (nm) *^c^*	Zeta Potential		TGA *^d^*
	*S*_BET_ (m^2^g^−1^)	*V*_pore_ (cm^3^g^−1^) *^a^*	∅ _pore_ (nm) *^b^*		pH	(mV)	
**E-P1** **90/10 PMO NPs**	973	0.66	2.7	1122	6.80	−30.9	77%
**E-P2** **90/10 PMO NPs**	696	0.37	2.1	694	6.72	−38.2	75%
**E-P1** **75/25 PMO NPs**	259	0.17	2.6	742	9.07	−25.6	67%
**E-P2** **75/25 PMO NPs**	329	0.23	2.7	562	6.85	−42.3	63%

*^a^* Determined by BET at saturation of p/p° = 0.98. *^b^* Pore diameter determined with BJH. *^c^* Hydrodynamic diameters, DLS. *^d^* Residual mass by TGA (heating rate 10 °C/min, 20 to 1000 °C).
